# Dynamic Changes in the Proteome of Early Bovine Embryos Developed *In Vivo*

**DOI:** 10.3389/fcell.2022.863700

**Published:** 2022-03-21

**Authors:** Charles Banliat, Coline Mahé, Régis Lavigne, Emmanuelle Com, Charles Pineau, Valérie Labas, Benoit Guyonnet, Pascal Mermillod, Marie Saint-Dizier

**Affiliations:** ^1^ CNRS, INRAE, Université de Tours, IFCE, UMR PRC, Nouzilly, France; ^2^ Union Evolution, Noyal-sur-Vilaine, France; ^3^ Irset–UMRS 1085, Inserm, University of Rennes, Rennes, France; ^4^ Protim, Univ Rennes, Biosit–UMS 3480, US-S 018, Rennes, France; ^5^ Pixanim, INRAE, Université de Tours, CHU de Tours, Nouzilly, France

**Keywords:** embryo, proteomics, mass spectrometry, morula, blastocyst, cattle, development

## Abstract

Early embryo development is a dynamic process involving important molecular and structural changes leading to the embryonic genome activation (EGA) and early cell lineage differentiation. Our aim was to elucidate proteomic changes in bovine embryos developed *in vivo*. Eleven females were used as embryo donors and pools of embryos at the 4–6 cell, 8–12 cell, morula, compact morula and blastocyst stages were analyzed by nanoliquid chromatography coupled with label free quantitative mass spectrometry. A total of 2,757 proteins were identified, of which 1,950 were quantitatively analyzed. Principal component analysis of data showed a clear separation of embryo pools according to their developmental stage. The hierarchical clustering of differentially abundant proteins evidenced a first cluster of 626 proteins that increased in abundance during development and a second cluster of 400 proteins that decreased in abundance during development, with most significant changes at the time of EGA and blastocyst formation. The main pathways and processes overrepresented among upregulated proteins were RNA metabolism, protein translation and ribosome biogenesis, whereas Golgi vesicle transport and protein processing in endoplasmic reticulum were overrepresented among downregulated proteins. The pairwise comparison between stages allowed us to identify specific protein interaction networks and metabolic pathways at the time of EGA, morula compaction and blastocyst formation. This is the first comprehensive study of proteome dynamics in non-rodent mammalian embryos developed *in vivo*. These data provide a number of protein candidates that will be useful for further mechanistic and functional studies.

## 1 Introduction

Mammalian embryo development starts after fertilization in the oviduct where the zygote undergoes its mitotic divisions while progressing toward the uterus. Bovine embryos stay in the oviduct for 3.5–4 days before they pass into the uterus, between the 8-cell and morula stages (Hamilton and Laing, 1946). During the first cleavage divisions, the embryo is transcriptionally quiescent, as its development is mainly controlled by oocyte-derived RNAs and proteins (Schulz and Harrison, 2019). Then, the zygotic genome is activated, leading to a progressive replacement of maternal and sperm supply by embryonic gene products (Lee et al., 2014). The major embryonic genome activation (EGA) event, also called maternal to zygotic transition (MZT), occurs between the 4-cell and 8-cell stages in humans (Braude et al., 1988) and around the 8-cell stage in cattle (Gad et al., 2012; Graf et al., 2014b; Jiang et al., 2014). Thereafter, the embryo becomes a morula, in which the blastomeres start to differentiate and bind firmly together in a process called compaction (Van Soom et al., 1997). In the compact morula, two distinct cell lineages differentiate to form a blastocyst: the inner cell mass, which will give rise to the embryo and the trophectoderm cells which will participate in the embryonic part of the placenta (Watson and Barcroft, 2001).

The molecular mechanisms governing mammalian embryo development are still poorly understood and, so far, very few studies have explored the dynamics in the proteome of the early embryo (Jensen et al., 2014a; Jensen et al., 2014b; Deutsch et al., 2014; Demant et al., 2015; Gao et al., 2017). The mouse model has been most widely used to study mammalian embryo development. However, bovine and human early embryos seem to be more similar in terms of biochemical regulatory processes, transcriptomic dynamics and the kinetics of development up to the blastocyst stage (Menezo and Herubel, 2002; Jiang et al., 2014; Schulz and Harrison, 2019), reinforcing interest in cattle embryos as models to study early development. Proteomic studies on cattle embryos have focused on a few developmental stages and were exclusively conducted with embryos produced *in vitro* (Jensen et al., 2014a; Jensen et al., 2014b; Deutsch et al., 2014; Demant et al., 2015), which are well-known to have divergent gene expression patterns (Lonergan et al., 2003; Gutierrez-Adan et al., 2004; Wrenzycki et al., 2005) and less developmental competence (Enright et al., 2000; Rizos et al., 2017) than their *in vivo* counterparts. The main reasons for this lack of information are the difficulty of accessing oviducts in live animals and the scarcity of the required material (Saint-Dizier et al., 2019).

Transcriptomic studies conducted on bovine embryos developed *in vivo* (Kues et al., 2008; Gad et al., 2012; Jiang et al., 2014; Diaz-Lundahl et al., 2021) or *in vitro* (Gad et al., 2012; Graf et al., 2014b; Kropp et al., 2017; Wei et al., 2017) have greatly expanded our comprehension of the regulatory mechanisms underlying the EGA, cell lineage specification and metabolic requirements during pre-implantation development. However, data on proteomic dynamics during early development *in vivo* are currently lacking in non-rodent mammals. The objectives of the present study were to identify for the first time dynamic changes in the proteome of bovine embryos developed *in vivo* from the 4–6 cell to blastocyst stages and to predict functional implication of differentially abundant proteins.

## 2 Results

**FIGURE 1:**
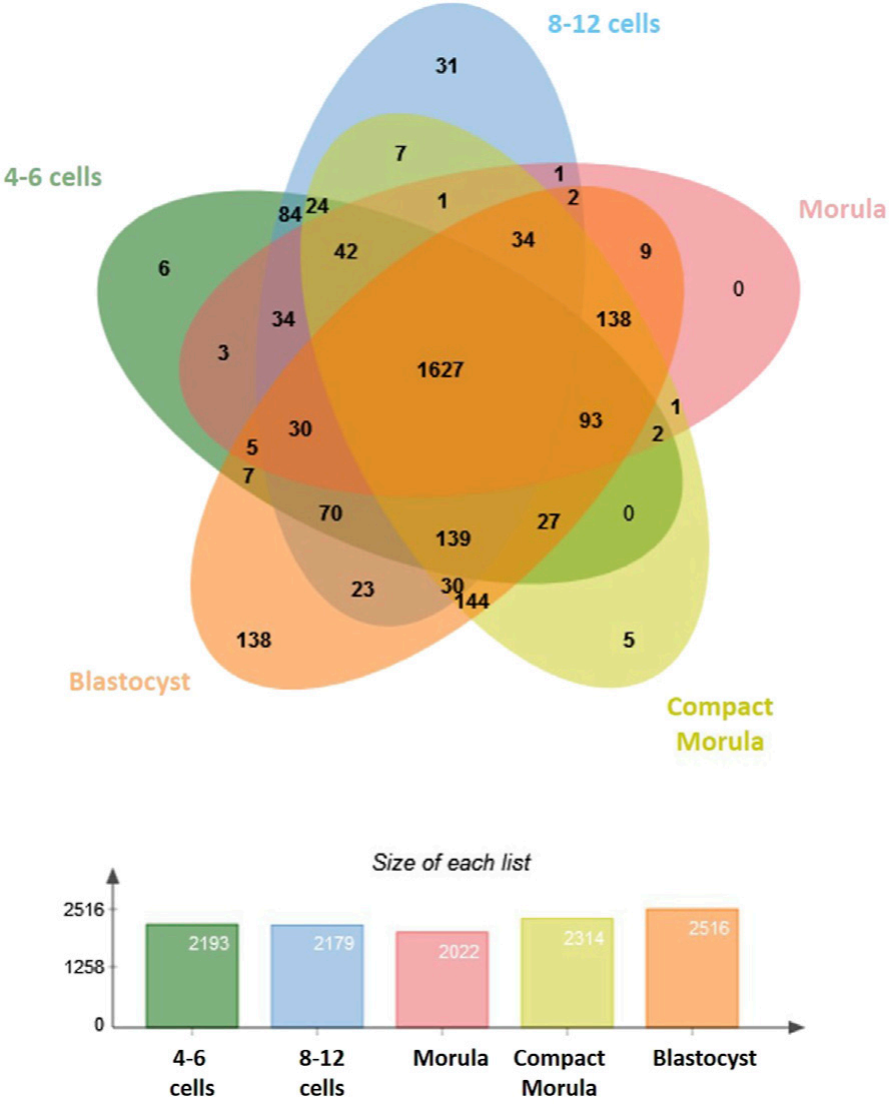
Comparative analysis of proteins identified in bovine early embryos. Venn diagram showing the overlap among developmental stages and histogram showing numbers of proteins identified in each stage.

In this study, we used nanoliquid chromatography coupled with tandem mass spectrometry (nanoLC-MS/MS) to elucidate dynamic changes in the proteome of bovine embryos developed *in vivo*. A total of 2,757 proteins were identified in embryos (see Supplementary Table S1 for the complete list of proteins with their accession number, gene symbol and normalized quantification). Overall, 1,627 identified proteins were shared between stages. The blastocyst stage displayed the highest number of specific proteins (138 specific proteins; Figure 1).

### 2.1 Global Analysis of Differentially Abundant Proteins Across Development

**FIGURE 2:**
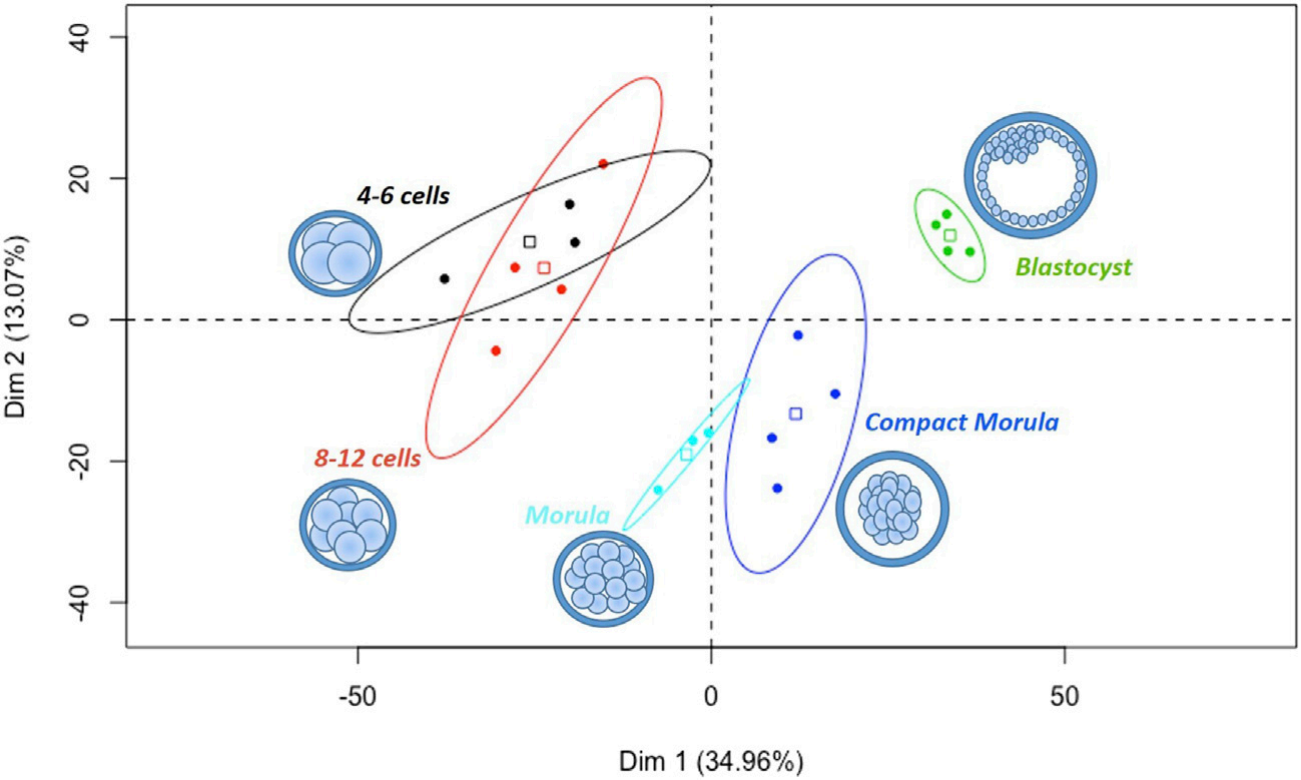
Principal component analysis of all *in vivo* embryos pools from the 4–6 cell to blastocyst stages. All proteins quantified with min 2 NWS in at least one stage were considered. Scatter plots represent the position of each pool of embryos along the first two dimensions of PCA. The variability between pools was mainly explained by their stage of development on the first horizontal dimension (Dim 1, 35% of variance). The square in each ellipse represents the mean of data for a given stage and colored ellipses represent the 95% confidence intervals.

A total of 1,950 proteins quantified with ≥2 normalized weighted spectra (NWS) in at least one condition were retained for statistical analysis (Supplementary Table S1). Oviduct-specific glycoprotein (OVGP1) was detected with high abundance (>100 NWS) at all stages. Principal component analysis (PCA) of all data showed a clear segregation of embryo pools according to their developmental stage, except between the 4–6 and 8–12 cell stages, which clustered together (Figure 2).

**FIGURE 3:**
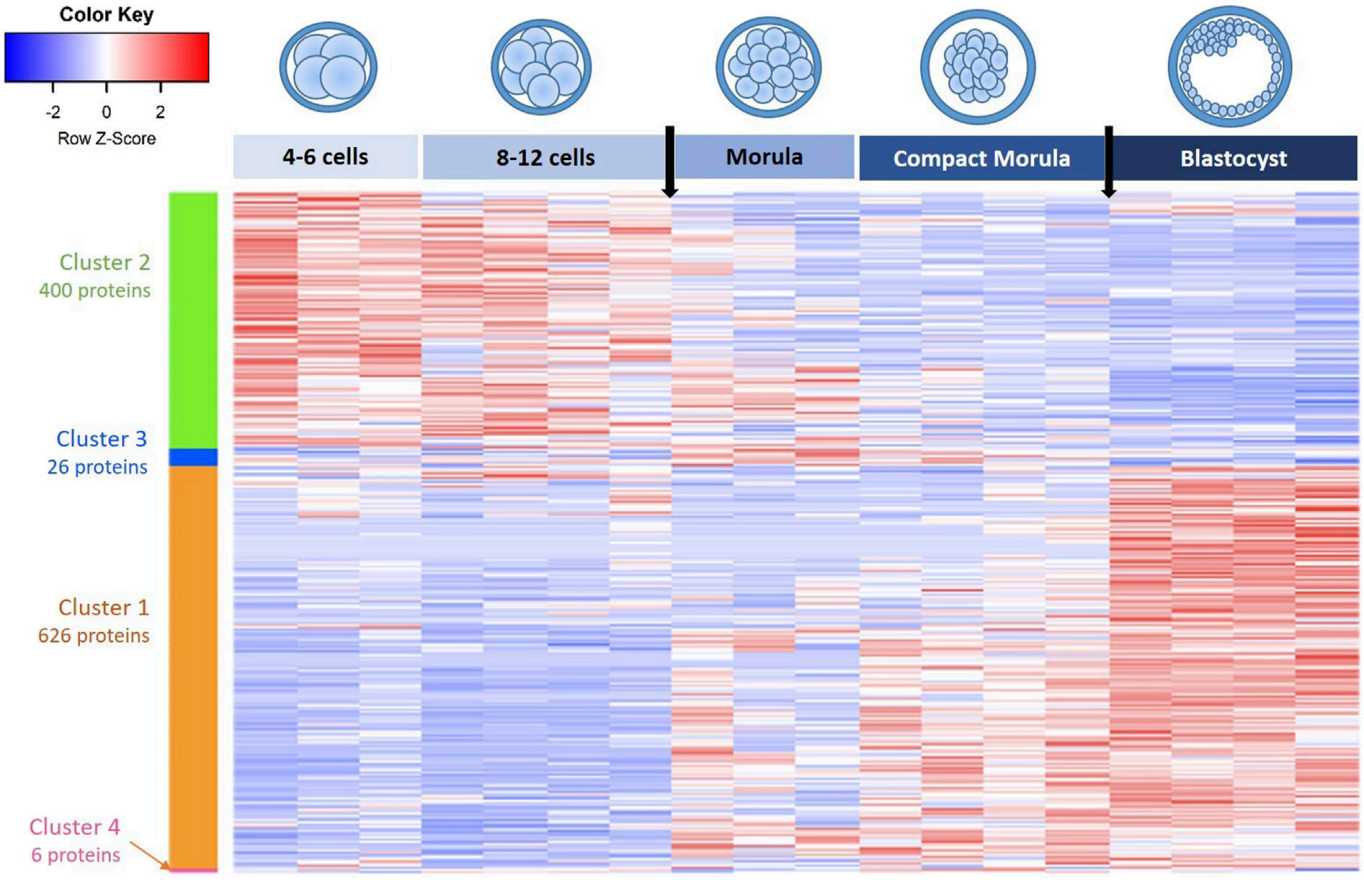
Heatmap and hierarchical clustering of differentially abundant proteins (DAPs) in *in vivo* embryos from the 4–6 cell to blastocyst stages. Each line corresponds to one protein and each row to the abundance values of one embryo pool. Red indicates higher abundance while blue indicates lower abundance compared with other conditions. Clusters of proteins are indicated by colored vertical bars and are numbered in the decreasing order of protein numbers. Black arrows on the top indicate biggest changes in protein abundance at the time of 8-12-cell-to morula and compact morula-to-blastocyst transitions.

The heatmap representation of differentially abundant proteins (DAPs) between stages (ANOVA p-value ≤ 0.05) evidenced that the biggest changes in protein abundance occurred between the 8–12 cell and morula stages then between the compact morula and blastocyst stages (vertical arrows in Figure 3).

The hierarchical clustering of DAPs allowed us to identity four clusters: a first cluster of 626 proteins that increased in abundance during development (upregulated proteins) and a second cluster of 400 proteins that decreased in abundance during development (downregulated proteins). Two other small clusters of 26 and 6 proteins, respectively, presented bimodal evolutions (see the lists of proteins in each cluster in Supplementary Table S2).

### 2.2 Functional Analysis of Differentially Abundant Proteins Across Development

**FIGURE 4:**
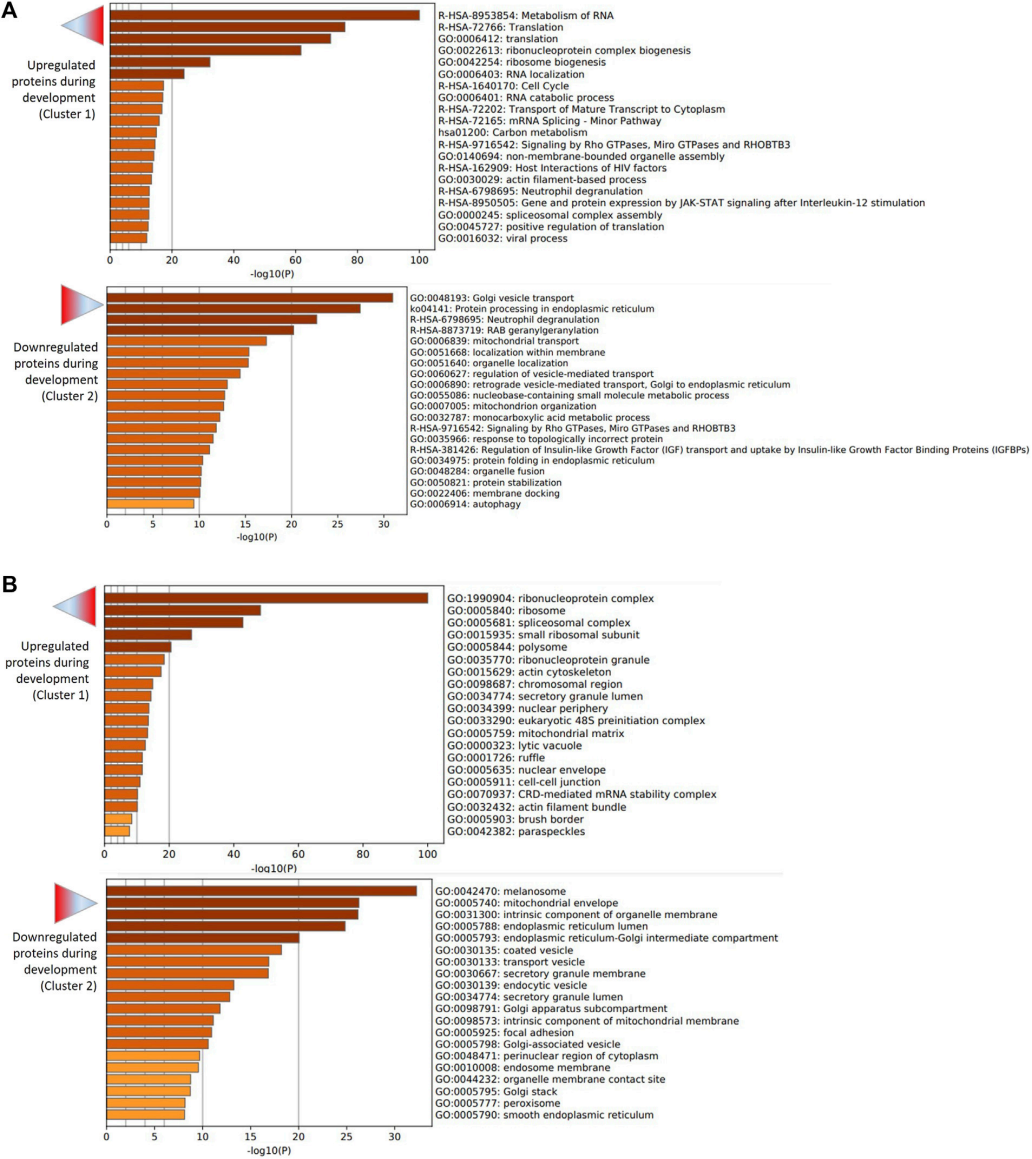
Functional enrichment analysis of upregulated (cluster 1) and downregulated (cluster 2) proteins during embryo development. The analysis was performed using Metascape. **(A)** Bar graph of enriched pathway and process terms for upregulated (top graph) and downregulated (bottom graph) proteins. **(B)** Bar graph of enriched cellular component terms for upregulated (top graph) and downregulated (bottom graph) proteins. Darker color of bars indicates higher significance (lower p-value).

The two first clusters were retained for functional analysis using Metascape. The enrichment analysis showed that pathways and processes related to the metabolism of RNA, protein translation (ribonucleoprotein complex biogenesis, ribosome biogenesis, RNA localization, RNA catabolic process) and carbon metabolism were overrepresented among upregulated proteins, whereas Golgi vesicle transport, protein processing in endoplasmic reticulum and neutrophil degranulation were overrepresented among downregulated proteins (Figure 4A and Supplementary Table S2 for all enriched GO terms according to Metascape). Furthermore, the enrichment analysis for cellular components (CC) showed that components of the protein translation machinery (ribonucleoprotein complex, ribosome, spliceosome complex, small ribosomal subunit, polysome …) were overrepresented among upregulated proteins. On the other side, proteins of the melanosome, mitochondrial envelope and intrinsic component of organelle membrane were overrepresented among downregulated proteins (Figure 4B; Supplementary Table S2).

### 2.3 Pair-Wise Comparisons Between Stages and Functional Implication

**FIGURE 5:**
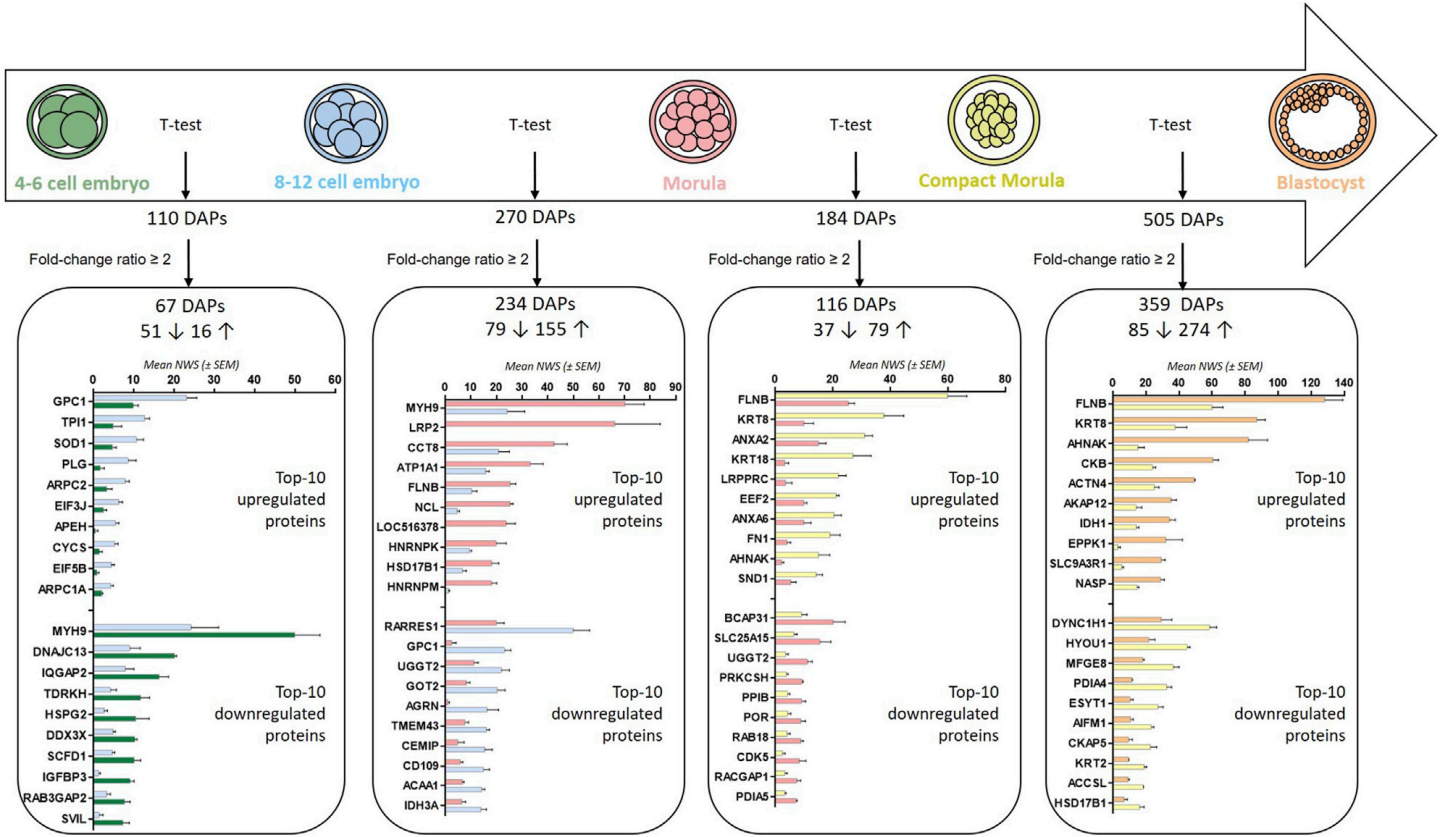
Numbers and top-20 differentially abundant proteins after pairwise comparisons between embryonic stages. Numbers of DAPs after t-tests (p-values ≤ 0.05) and considering a min fold-change ratio of 2 are presented. Numbers before rising and downward arrows indicate upregulated and downregulated proteins, respectively. The histograms indicate the top-10 upregulated and downregulated proteins in each comparison.

Pairwise comparisons (t-test p-value ≤ 0.05) between stages evidenced between 110 and 505 DAPs in total and between 67 and 359 DAPs considering a minimal fold-change of 2 (Figure 5; see Supplementary Table S3 for complete lists of DAPs after t-tests). In accordance with the heatmap on Figure 3, the highest numbers of DAPs were observed at the 8–12 cell-to-morula and compact morula-to-blastocyst transitions.

DAPs with a fold-change ratio ≥2 were further studied for their functional involvement and interaction networks. Between the 4–6 and 8–12 cell stages, due to the relatively low number of DAPs, no PPI network and only two enriched KEGG pathways were identified (RNA transport and regulation of actin cytoskeleton; p-values ≤ 0.05; see enriched terms during the 4–6-to-8–12 cell transition in Supplementary Table S4).

#### 2.3.1 From 8–12 Cell to Morula Stages

**FIGURE 6:**
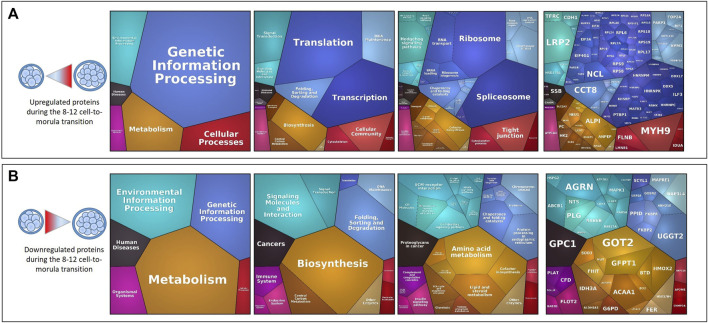
Proteomaps of differentially abundant proteins (DAPs) during the 8–12 cell-to-morula transition. Proteomaps were carried out using the lists of gene names of proteins increasing [**(A)**, upper panels] or decreasing [**(B)**, down panels] in abundance (t-test p-value ≤ 0.05; min fold-change of 2) between the 8–12 cell and morula stages and based on the KEGG Pathway gene classification. Functional categories (left and middle panels) and related proteins (right panel) are shown by polygons. Areas of polygons illustrate protein abundance, weighted by protein size. Functionally related functions/proteins are arranged in common regions and coded using similar colors.

From the 8–12 cell to morula stages, the Proteomaps of DAPs showed that upregulated proteins were mainly involved in the transcription and translation pathways (ribosome, RNA transport, spliceosome) and to a lesser extent in cellular processes (cellular community, cytoskeleton) and biosynthesis (Figure 6A). On the other hand, downregulated proteins were overrepresented among the environmental information processing category (ECM-receptor interactions, CD molecules, GTP-signaling molecules) and metabolic pathways (amino acid metabolism, lipid and steroid biosynthesis, cofactor biosynthesis and carbon metabolism) (Figure 6B).

**FIGURE 7:**
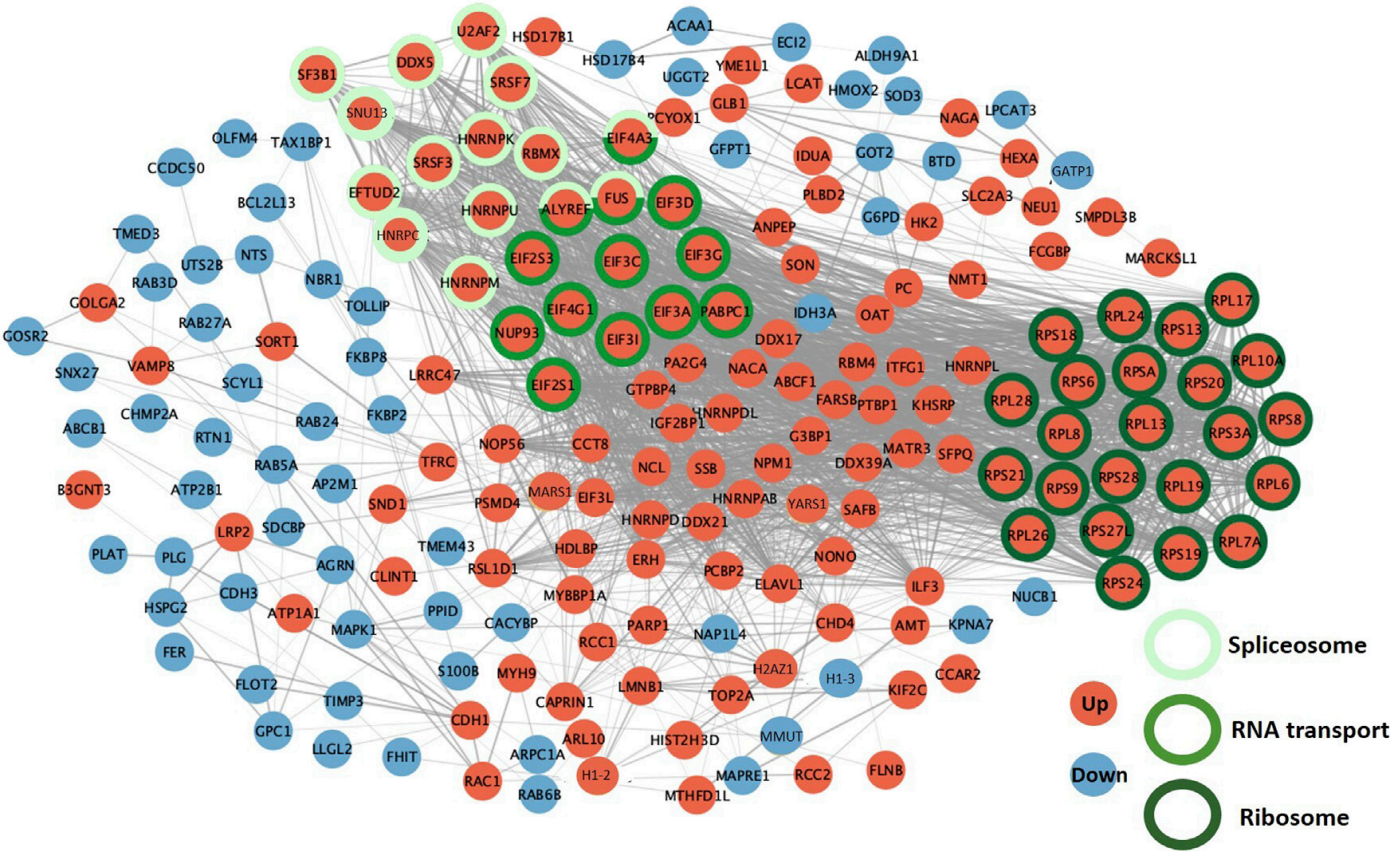
Protein-protein interaction networks between differentially abundant proteins (DAPs) during the 8–12-cell-to-morula transition. The PPI network was built using STRING and Cytoscape. DAPs increased in abundance between the 8–12 cell and morula stages are in red while proteins decreased in abundance are in blue. The DAPs involved in the three most significant KEGG pathways are indicated by colored circles in green.

The STRING (Search Tool for the Retrieval of Interacting Genes/proteins) functional analysis of DAPs during the 8–12 cell-to-morula transition confirmed the highly significant enrichment in the ribosome, spliceosome and RNA transport pathways (p-values < 10^−5^; see all enriched terms during the 8–12 cell-to-morula transition in Supplementary Table S5). In addition, numerous protein interactions were identified, including two dense PPI networks encompassing several ribosomal proteins and proteins involved in RNA transport and the spliceosome that were all upregulated (Figure 7; see network details in Supplementary Table S5).

#### 2.3.2 From Morula to Compact Morula Stages

**FIGURE 8:**
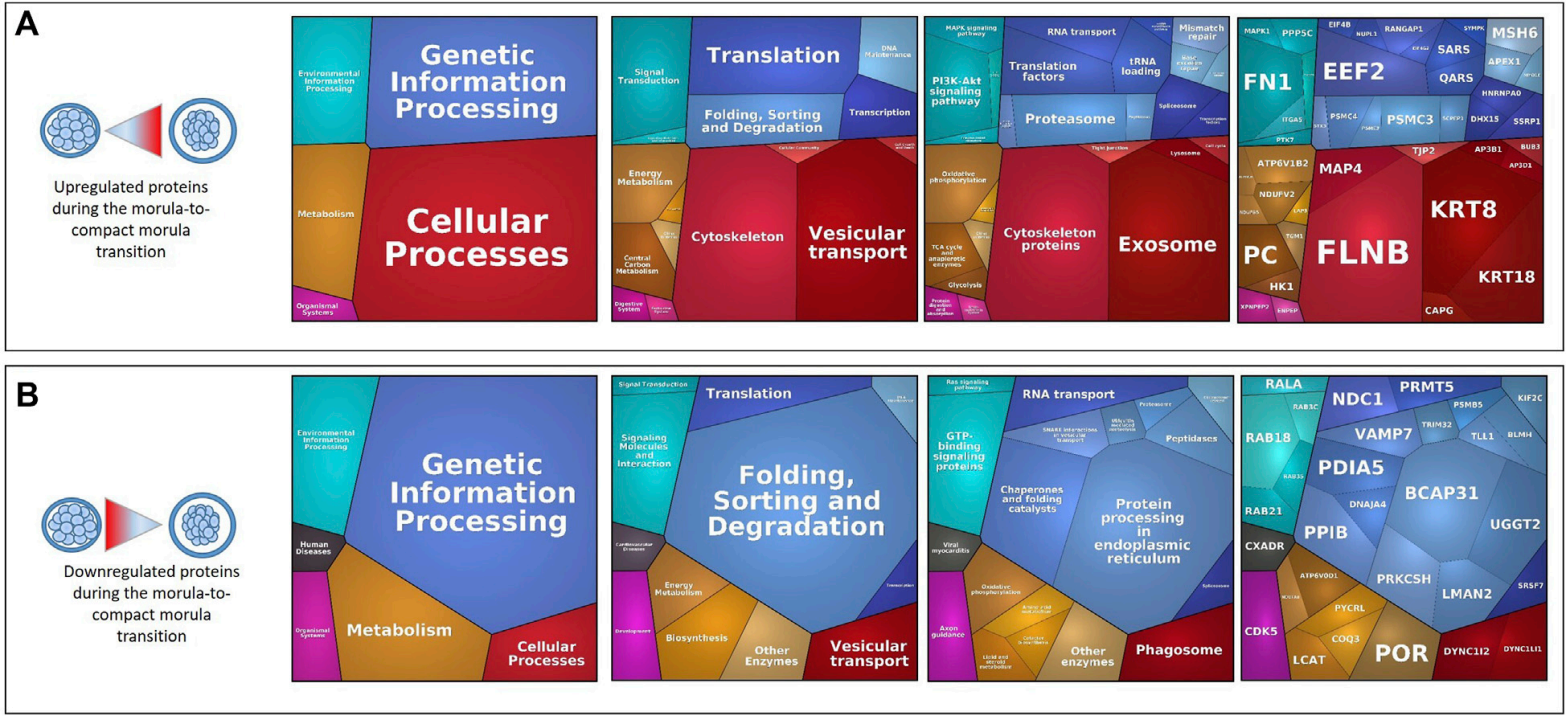
Proteomaps of differentially abundant proteins (DAPs) during the morula-to-compact morula transition. Proteomaps were carried out using the lists of gene names of proteins increasing [**(A)**, upper panels] or decreasing [**(B)**, down panels] in abundance (t-test p-value ≤ 0.05; min fold-change of 2) between the morula and compact stages and based on the KEGG Pathway gene classification. Functional categories (left and middle panels) and related proteins (right panel) are shown by polygons. Areas of polygons illustrate protein abundance, weighted by protein size. Functionally related functions/proteins are arranged in common regions and coded using similar colors.

Between the morula and compact morula stages, Proteomaps of DAPs showed that upregulated proteins were mainly involved in the cytoskeleton and vesicular transport pathways, whereas downregulated proteins were largely involved in protein folding, sorting and degradation (Figure 8). The STRING analysis did not identify enriched pathways but several enriched molecular functions (MF) and biological process (BP) terms including developmental process and multicellular organism development (see all enriched terms and the PPI network during morula compaction in Supplementary Table S6).

#### 2.3.3 From Compact Morula to Blastocyst Stages

**FIGURE 9:**
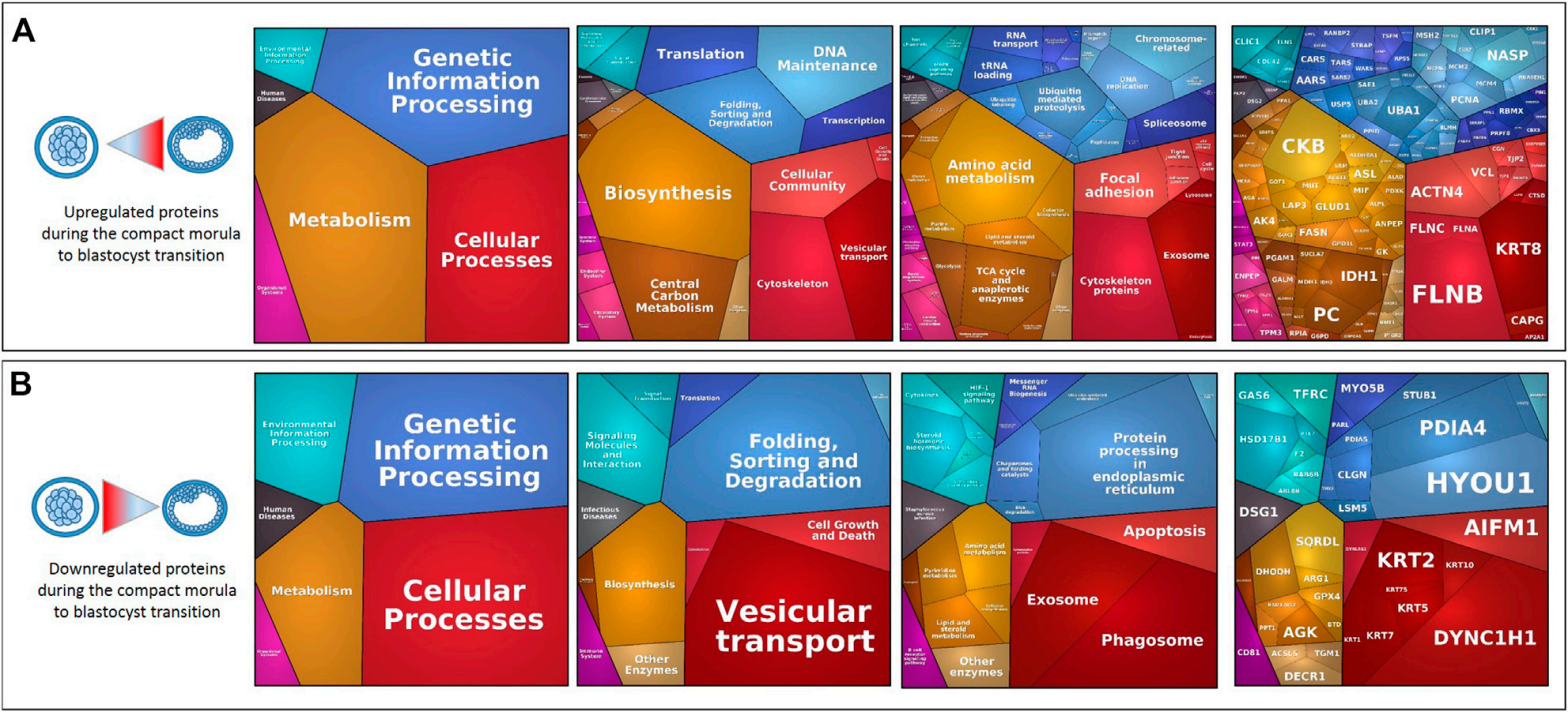
Proteomaps of differentially abundant proteins (DAPs) during the compact morula-to-blastocyst transition. Proteomaps were carried out using the lists of gene names of proteins increasing [**(A)**, upper panels] or decreasing [**(B)**, down panels] in abundance (t-test p-value ≤ 0.05; min fold-change of 2) between the compact morula and blastocyst stages and based on the KEGG Pathway gene classification. Functional categories (left and middle panels) and related proteins (right panel) are shown by polygons. Areas of polygons illustrate protein abundance, weighted by protein size. Functionally related functions/proteins are arranged in common regions and coded using similar colors.

**FIGURE 10:**
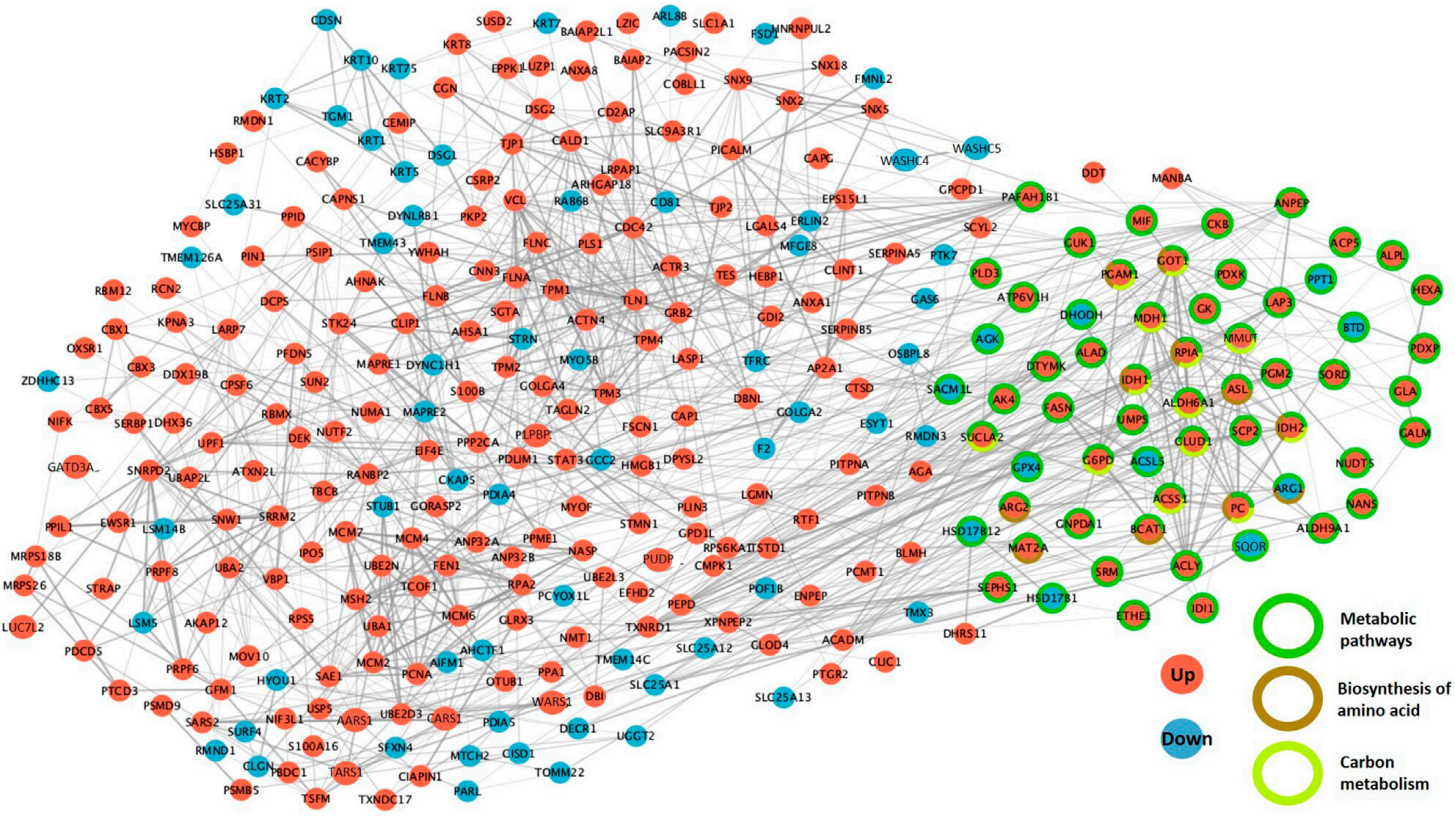
Protein-protein interaction networks between differentially abundant proteins (DAPs) during the compact morula-to-blastocyst transition. The PPI network was built using STRING and Cytoscape. DAPs increased in abundance between the compact morula and blastocyst stages are in red while proteins decreased in abundance are in blue. The DAPs involved in the three most significant KEGG pathways are indicated by colored circles in green, brown and yellow.

Between the compact morula and blastocyst stages, upregulated proteins related to cellular processes were involved in the cytoskeleton, vesicular transport and cellular community pathways whereas downregulated proteins were more specifically involved in the apoptosis, exosome and phagosome pathways. Concerning genetic information processing pathways, upregulated proteins were involved in transcription, translation and DNA maintenance while downregulated proteins were mainly involved in protein folding, sorting and degradation. In addition, both up and downregulated proteins were involved in metabolic pathways such as amino acid metabolism and lipid and steroid metabolism, however upregulated proteins were more specifically involved in glycolysis and tricarboxylic acid (TCA) cycle (Figure 9). The STRING enrichment analysis of DAPs at the time of blastocyst formation confirmed highly significant enrichment in metabolic pathway, carbon metabolism, biosynthesis of amino acids (p-values < 10^−4^) and TCA cycle (*p* = 0.003; see all enriched terms during blastocyst formation in Supplementary Table S7). Finally, numerous interactions between DAPs were identified including proteins implied in the above metabolic pathways (Figure 10; see network details in Supplementary Table S7).

## 3 Discussion

Very few studies on embryonic proteomes have been reported, and the large majority of them used *in vitro* embryos (Jensen et al., 2014a; Jensen et al., 2014b; Deutsch et al., 2014; Demant et al., 2015; Gao et al., 2017), which may give a biased picture of embryo protein dynamics due to divergent gene expression (Lonergan et al., 2003; Gutierrez-Adan et al., 2004; Wrenzycki et al., 2005). The present study identified 2,757 proteins in bovine embryos developed *in vivo*, representing the most comprehensive embryo proteome identified in non-rodent mammals so far.

Interestingly, oviduct-specific glycoprotein (OVGP1), also known as oviductin, was detected among the most abundant proteins at all stages. Oviductin was identified in the proteomic profile of ovine blastocyst developed *in vivo* (Sanchez et al., 2021) and as a major embryo-interacting protein in the bovine oviduct fluid (Banliat et al., 2020). Oviductin has been reported to interact with embryos in various mammalian species (Aviles et al., 2010) and improves the quality of *in vitro* produced cattle embryos when added as a recombinant protein in culture medium (Algarra et al., 2018).

### 3.1 Proteomic Dynamics During Embryonic Genome Activation

The number of proteins identified per embryo slightly decreased from the 4–6 cell to morula (2,193 to 2,022) then increased from compact morula to blastocyst stages (2,314 to 2,516). Upregulated proteins (cluster 1) from the 4–6 cell to the blastocyst stages evidenced a significant enrichment in RNA metabolism and protein translation, presumably to provide enough materials to support further development. This is globally in accordance with previous data on protein synthesis in bovine embryo that indicated a higher rate of protein degradation than that of synthesis in early stages, the net protein growth per embryo starting only after morula compaction (Thompson et al., 1998). In line with our data, upregulated proteins during *in vivo* development of mouse embryos tended to execute protein translation and RNA metabolism while downregulated proteins were mostly involved in protein transport, protein localization and cell cycle (Gao et al., 2017). This is also concordant with the timing of EGA during which maternal proteins, and to a lesser extent paternal proteins, are progressively replaced by embryonic gene products (Graf et al., 2014a).

The onset of EGA in cattle embryos was established according to transcriptomic data with some discrepancies between studies. Using *in vitro* embryos, the onset of major EGA was timed between the 8 and 16-cell stages (Memili and First, 1998; Graf et al., 2014b); however, transcriptomic analyses of *in vivo* embryos reported an EGA just before the 8-cell stage (Kues et al., 2008; Jiang et al., 2014). Our results provide for the first time a comprehensive picture of proteomic dynamics during the EGA. Major changes in the dynamics of protein synthesis were first evidenced after the 8–12 cell stage, which is consistent with a major EGA at the 8-cell stage. Due to the collection method, the donor cows of morulas differed from those of 8–12 cell embryos, which may account for changes in proteomic dynamics. Nevertheless, upregulated proteins during the 8–12-cell-to-morula transition evidenced a significant enrichment in functional pathways and protein-protein interaction networks related to transcription, RNA transport, RNA splicing and protein translation, which are assumed to underlie the EGA.

### 3.2 Proteomic Dynamics and Functional Candidates During Morula Compaction

To the best of our knowledge, this is the first proteomic study to separately analyze morulas and compact morulas in mammals, providing insights into the molecular mechanisms underlying the compaction process. Compaction marks the start of important cell differentiation events: the outer blastomeres of the morula adhere firmly to each other and give rise to the trophectoderm, while the inner blastomeres of the compact morula form the inner cell mass of the blastocyst (Van Soom et al., 1997; Watson and Barcroft, 2001). A total of 184 DAPs were identified at the time of morula compaction, of which 116 DAPs with a minimal fold-change ratio of 2. In contrast to the 8-cell-to-morula transition, during which genetic information processing pathways were predominant, the Proteomaps of DAPs during morula compaction showed that upregulated proteins were largely involved in cellular processes, in line with important cell differentiation events during this process.

The cellular process-related proteins fibronectin 1 (FN1), filamin B (FLNB), keratins 8 (KRT8) and 18 (KRT18) were particularly increased during compaction (fold-changes of 4.6, 2.4, 3.8 and 7.9, respectively). Fibronectin 1 is a large glycoprotein of the extracellular matrix that binds cell surfaces and is involved in a variety of biological processes including cell adhesion, cell migration and the maintenance of cell shape (Humphries et al., 1989). Previous transcriptomic data on bovine *in vivo* embryos reported a marked increase in FN1 transcripts between the 8-cell and blastocyst stages (Goossens et al., 2007; Goossens et al., 2009) and immunolabelling for FN1 on *in vitro* produced embryos was first observed in late morulas (Goossens et al., 2007), in accordance with our results. The assembly of FN1 with integrin partners on neighboring cells were shown to play important roles in tissue differentiation during mouse and chicken embryo development (de Almeida et al., 2016). In cattle embryos, several potential integrin partners were reported to have similar or overlapping RNA expression patterns as compared to FN1 (Goossens et al., 2009); however, the exact candidate receptors of FN1 in morulas remain to be identified.

The filamin family of cytoplasmic actin-binding proteins exert crucial signaling functions in cell cytoskeleton dynamics and tissue morphogenesis during embryo development (Baldassarre et al., 2009; Baudier et al., 2018). In mice with a targeted disruption of the *Flnb* gene, less than 3% of homozygous embryos reached term, and the migration of primary fibroblasts was reduced, indicating a pivotal role of filamin B in embryonic development (Zhou et al., 2007). In various human cell lines, filamin B was also identified as a key regulator of cell migration (Baldassarre et al., 2009; Del Valle-Perez et al., 2010). The exact roles of fibronectin 1 and filamin B in cattle early embryo development remain to be determined but their abundance pattern together with previous published data strengthen the idea that those proteins are involved in the compaction of morula leading to cell lineage differentiation.

Other interesting candidates are keratin 8 and 18. Keratin proteins constitute the intermediate filaments of epithelial cells and were expressed by preimplantation embryos in several species (Oshima et al., 1983; Sun et al., 2010). In our analysis, we excluded human keratin contaminants by comparing the sequences of the MS peptides identified with their homologous in *Homo sapien*s. Upregulation of KRT8 and 18 during compaction were in line with previous transcriptomic data on bovine embryos, in which gene expression of *KRT8* and *KRT18* was greatly increased between morula and blastocyst stages (Goossens et al., 2007; Wei et al., 2017). Using single cell gene expression analysis, *KRT8* was reported to display a bimodal distribution among cells in bovine morulas, suggesting that KRT8 is an early marker of trophectoderm cells during early lineage specification (Wei et al., 2017). In addition, the protein expression of KRT18 has been reported to start at the morula stage and to be specific to the trophectoderm in bovine embryos produced *in vitro* (Goossens et al., 2007). Suppression of *KRT18* gene expression in bovine zygotes led to an associated reduction in *KRT8* expression and a 41-% reduction in blastocyst formation (Goossens et al., 2010). Our results, together with those of previous studies, suggest important roles for KRT8 and KRT18 in preimplantation bovine embryos.

Another upregulated protein during morula compaction and blastocyst formation (with fold-changes of 2.1 and 1.8, respectively) was elongation factor 2 (EEF2). EEF2 catalyzes the GTP-dependent ribosomal translocation step during translation elongation and is an essential factor for protein synthesis. *EEF2* was previously identified in a transcriptomic study in bovine *in vivo* blastocysts as a hub gene, i.e. central and highly connected with other stage-specific regulated genes, and as such was proposed as a master regulator of gene expression during pre-implantation development (Jiang et al., 2014).

### 3.3 Proteomic Dynamics and Functional Candidates During Blastocyst Formation

Another major change in protein dynamics was evidenced at the compact morula-to-blastocyst transition, which demonstrated the highest number of DAPs: among these, 359 had fold-change ratios greater than 2. Donors of blastocysts partly differed from those of compact morulas and included both Holstein cows and heifers. Furthermore, three bulls of different breeds (one Normande and two Holsteins) were used for blastocysts while only the Normande bull was the progenitor of all other embryos, which may account for the specificities of blastocysts in the present study. It has been shown that the father has an impact on gene expression and epigenomics in bovine blastocysts (Kropp et al., 2017; Diaz-Lundahl et al., 2021). However, the fertility of the bulls used, as previously assessed on a large population of heifers and cows, was high and homogeneous (59–62% and 45–49% of non-return rates at 90 days after insemination, respectively). Furthermore, the different pools of blastocysts had very similar proteomic profiles, pointing out a major effect of the developmental stage rather than the genetic origin of progenitors or female parity on the embryo proteome. In a previous proteomic study comparing six stages of *in vivo* mice embryos derived from a much higher number of progenitors (8000 embryos per stage), the most significant changes in protein abundance were similarly observed between morulas and blastocysts (Gao et al., 2017). These data highlight the specificity of the protein expression profiles in blastocysts compared to earlier developmental stages.

Considering a minimal fold-change ratio of two, 274 proteins were found at significantly higher abundance in blastocysts compared to compact morulas, which represented 76% of the total number of DAPs. Similarly, 102 proteins out of 140 DAPs (73%) were found to be increased in abundance between cattle *in vitro* morulas and blastocysts (Demant et al., 2015). This high proportion of upregulated proteins is in line with the overrepresented translation-related pathways observed in earlier stages and the increasing need for materials during embryo development.

The most abundant upregulated proteins included annexins A2 and A6 (ANXA2, ANXA6), AHNAK nucleoprotein (AHNAK), creatine kinase B (CKB), alpha-actin-1 and 4 (ACTN1, ACTN4), LIM and SH3 domain protein 1 (LASP1), and fibronectin 1 (FN1), whereas hypoxia up-regulated protein 1 (HYOU1), lactadherin (MFGE8), and protein disulfide-isomerase A4 and A5 (PDIA4, PDIA5) were at lower abundance in blastocysts compared to compact morulas. Our results are partially consistent with a previous proteomic study that compared cattle *in vitro* morulas and blastocysts, in which ANXA2, ANXA6, CKB, ACTN1 and LASP1 were upregulated while HYOU1, PDIA4 and PDIA5, among others, were downregulated (Demant et al., 2015). In addition, some upregulated proteins (cluster 1) like the transketolase (TKT), myosin light polypeptide 6 (MYL6), 60S ribosomal protein L13 (RPL13) and D-dopachrome decarboxylase (DDT) increased also in abundance in a study by Demant et al. (2015). However, some discrepancies between both studies were also found. For instance, phosphoglycerate mutase (BPGM), L-lactate dehydrogenase A (LDHA), glutathione S-transferase mu 3 (GSTM3), ubiquitin carboxyl-terminal hydrolase isozyme L1 (UCHL1) and peroxiredoxin-1 (PRDX1) were stable in abundance during embryo development in the present study, but were identified among the top-25 downregulated proteins during blastocyst formation in the study by Demant et al. (2015). The *in vitro* or *in vivo* origin of embryos and the methods of analysis (iTRAQ *vs*. label-free MS approaches) may account for those discrepancies.

DAPs during blastocyst formation may be considered as early markers of cell lineage differentiation and important functional candidates for the regulation of key developmental processes. Keratin 8 and filamin B were both upregulated (fold change >2) during morula compaction and in blastocysts, in which they were among the most abundant DAPs. Another interesting candidate protein is AHNAK, with fold-changes of 6.5 and 5.8 during morula-to-compact morula and compact morula-to-blastocyst transitions, respectively. AHNAKs are a class of unusually large proteins (600–700 kDa) that associate with calcium channel proteins and have various subcellular localization depending on the cell type and cell cycle progression (Komuro et al., 2004). During mouse development, AHNAK protein expression was found in derivatives of trophectoderm and in several major sites of placentation and neurulation, suggesting multiple developmental roles (Downs et al., 2002). However, functional studies on AHNAK during earlier stages of embryo development are currently lacking.

Annexins 1, 2, 6 and 8 are other potential stage-specific markers of embryonic cell differentiation: ANXA2 and ANXA6 were detected at increased abundance during morula compaction (with fold-changes of 2.0 and 2.1, respectively), while ANXA1, ANXA6, and ANXA8 were increased during blastocyst formation (with fold-changes of 3.4, 1.8 and 5.3, respectively). Annexins are a family of calcium- and phospholipid-binding proteins bearing multiple functions and that could play important roles during the process of blastocyst differentiation. Annexins 1 and 2 have been previously reported in the blastocyst cells and blastocoel fluid of *in vitro* produced bovine embryos (Jensen et al., 2014b). Annexin 1 is involved in cell adhesion and promotes the rearrangement of the actin cytoskeleton, cell polarization and cell migration in immune cells (Moraes et al., 2017). ANXA1 has been linked to pregnancy in mice: *AnxA1*-deficient females presented increased numbers of blastocysts, implantation sites and pups delivered (Hebeda et al., 2018). In humans and mice, ANXA2 is transiently expressed at the embryo-uterine luminal interface and has been implicated as an important adhesion molecule in the process of embryo implantation (Garrido-Gomez et al., 2012; Wang and Shao, 2020). The development of cattle embryos differs from that of humans and mice in that their trophectoderm elongates in the uterine lumen before they finally attach to the endometrium, and such roles of annexin A2 in embryo attachment lacks evidence so far in this species.

### 3.4 Metabolic Pathways Activated During Early Embryo Development

The GO analysis of DAPs at the time of blastocyst formation evidenced a significant enrichment in metabolic pathways, carbon metabolism, biosynthesis of amino acids and the TCA cycle. The well-orchestrated activation of metabolic pathways is required during early embryo development. During the first cleavage divisions, mammalian embryos, including bovine, use mostly pyruvate and amino acids as preferred substrates, which are metabolized through the TCA cycle and oxidative phosphorylation to produce energy (Khurana and Niemann, 2000; Li and Winuthayanon, 2017). At the time of compaction, the demand for energy and biomass increases and glucose is metabolized with greater efficiency through the pentose phosphate pathway (PPP), important for the biosynthesis of nucleotides, and the glycolysis pathway in addition to the TCA cycle (Milazzotto et al., 2020). Furthermore, substrates generated by metabolism seem to be important players in epigenetic modifications in preimplantation embryos (Ispada et al., 2020; Milazzotto et al., 2020).

Based on biochemical studies in cattle embryos developed *in vivo*, there is evidence that compaction and blastocyst formation are accompanied by marked increases in the consumption of glucose (Khurana and Niemann, 2000). This is globally in line with the proteomic dynamics observed in our results. Several enzymes implied in the production of energy and biomass from glucose were upregulated and identified in the first cluster of DAPs across development. This was the case for glucose-6-phosphate 1-dehydrogenase (G6PD), which catalyzes the first rate-limiting step of the PPP, 6-phosphogluconate dehydrogenase (PGD), ribose-5-phosphate isomerase (RPIA), transketolase (TKT) and transaldolase (TALDO1) in the PPP. This was also the case for hexokinase (HK2), implied in the first step of glycolysis, phosphoglycerate kinase 1 (PGK1) and phosphoglycerate mutase 1 (PGAM1) in the glycolysis pathway. In particular, the protein abundance of HK1 and HK2 significantly increased during morula compaction with fold-changes of 10.4 and 1.6, respectively, while after compaction during blastocyst formation, G6PD, TALDO1, PGAM1 and PGK1 increased with fold-changes of 5.0, 1.9, 2.8, and 1.8, respectively. However, some other glycolytic enzymes like fructose-bisphosphate aldolase (ALDOC) and glyceraldehyde-3-phosphate dehydrogenase (GAPDH) decreased in abundance, although with lower fold-changes (1.7 and 1.4, respectively), during blastocyst formation. These data confirm a global switch in the energy requirements of the embryo toward more glucose after the major EGA and constitute an important step forward for the comprehension of embryo metabolism and regulation during *in vivo* mammalian development.

### 3.5 Limitations of the Study

One limitation of the present study is that the embryos were collected after estrus synchronization and superovulation treatment. This was done for evident ethical, technical and economic reasons. Superovulation represents the primary tool for the production of cattle embryos and has been commercially practiced for embryo transfer on a wide scale for more than 40 years (Seidel, 1981). However, circulating concentrations of reproductive hormones such as progesterone can be modified in superovulated cows and heifers (Lamond and Gaddy, 1972), and an effect of this particular endocrine environment on the embryo proteome cannot be excluded. As discussed earlier, another weakness compared to studies in mice is the relative low number of animals and higher heterogeneity between progenitors.

Another limitation is that, due to the required materials for MS analysis, the use of extra *in vivo* embryos to explore changes in candidate proteins by complementary methods was not possible. Due to the divergence in gene expression reported between bovine embryos developed *in vivo* or *in vitro* (Lonergan et al., 2003; Gutierrez-Adan et al., 2004; Wrenzycki et al., 2005), the use of *in vitro* embryos for protein validation was not judged to be suitable.

## 4 Conclusion

This is the first comprehensive evaluation of proteome dynamics during early bovine development *in vivo*. Our results indicate significant changes in protein dynamics at the time of EGA and during first cell lineage differentiation during morula compaction and blastocyst formation. Here, we provide a large database of new protein markers of these processes and valuable candidates that make possible further functional studies on embryo development. These data may also be used to evaluate the impact of various *in vitro* conditions on the embryo proteomics and improve the safety and yield of embryo culture media.

## 5 Materials and Methods

### 5.1 Collection and Preparation of *In Vivo* Embryos From Synchronized Donors

All experimental protocols were conducted following the European directive 2010/63/EU on the protection of animals used for scientific purposes and approved by the French Ministry of National Education, Higher Education, Research and Innovation after ethical assessment by the local ethics committee “Comité d’Ethique en Experimentation Animale Val de Loire (CEEA VdL)” (protocol registered under the number CE19-2021-0402-1).

A total of 11 Holstein females (9 dried off cows and 2 heifers, aged 1–5 years) housed at the INRAE Experimental Unit of Animal Physiology of the Orfrasière (Nouzilly, France) were used as donor animals. The estrus cycles of the donors were synchronized with the prostaglandin analog cloprostenol (0.5 mg, i.m.; Estrumate, MSD Animal Health, Beaucouzé, France) and all females showing normal estrus, following an ovarian corpus luteum 1 week later (as checked by ultrasonography), received a progesterone releasing intravaginal device (PRID DELTA, Ceva Santé animale, Libourne, France). Ovarian stimulation treatment started 12 days after estrus and consisted of eigth intramuscular injections of decreasing pFSH/pLH doses every 12 h over 4 days (Stimufol, Reprobiol, Liège, Belgique; 500 µg pFSH and 100 µg LH in total in cows; 350 µg pFSH and 70 µg pLH in total in heifers). Luteolysis was induced with 2 ml cloprostenol (0.5 mg, i.m; Estrumate, MSD Animal Health, Beaucouzé, France) and the PRID DELTA device was withdrawn together with the fourth and sixth FSH administrations, respectively.

For embryo collection, donor females were inseminated with frozen-thawed semen from one bull of proven fertility 12 and 24 h after standing estrus. The same ejaculate from one Normande bull (bull X) was used for most embryos while two extra Holstein bulls (Y and Z) were used for blastocysts (Table 1). The fertility of the bulls was previously estimated by the 90-day non-return rate after artificial insemination on a large population of dairy females and ranged from 59 to 62% in heifers (3,241–8,587 AI per bull) and from 45 to 49% in lactating cows (22,864–26,623 AI per bull). Early stages (from 4–6 cell to compact morula) and 6 blastocysts were recovered after the slaughter of donor cows between days 1.7 and 7.5 after the first AI (AI1) in a local experimental slaughterhouse. The genital tracts were immediately processed on site. Oviducts and uterine horns were flushed with 5 and 35 ml, respectively, of prewarmed PBS+0.1% polyvinyl alcohol (PVA) at 38.5°C. Additional blastocysts were collected by non-invasive cervical flushing of the uterine horns of heifers on day 7 after AI1, as previously described (Janati Idrissi et al., 2021). Recovered fluids were carefully observed for the presence of embryos under a stereomicroscope and all embryos were classified for quality grade and stage of development according to the International Embryo Technology Society recommendations (Wright, 1998). Representative pictures of embryos are shown in Supplementary Figure S1.

**TABLE 1:** Pools of *in vivo* produced bovine embryos used for nanoLC-MS/MS analysis (*n* = 4 embryos per pool).

Embryonic stage	Interval of embryo collection after first insemination	Female donor^a^	Bull semen^b^	Number of pools
4–6 cells	Days 1.7–3.8	A, B, C, D	X	3
8–12 cells	Days 3.6–3.8	A, B	X	4
Morula (>16 cells)	Days 5.7–6.7	E, F, G	X	3
Compact morula	Days 5.7–6.8	E, F, H	X	4
Blastocyst	Days 6.8–7.5	H, I, J, K	X, Y, Z	4

a A total of 9 Holstein cows (called A to I) and 2 Holstein heifers (called J and K) were used as female donors.

b A total of 3 bulls of proven fertility, one Normand (called X) and two Holstein (called Y and Z), were used for the insemination of female donors.

All embryos were thoroughly washed three times in 20 mM Tris-HCl buffer (pH 6.8) supplemented with 8.9% sucrose (Tris-sucrose), minimizing contamination from oviduct ad uterine fluids, then individually frozen at −80°C. Typically, the time period between animal death and genital tract flushing and between flushing and embryo freezing was less than 15 and 30 min, respectively. For proteomic analyses, only grade-1 embryos, i.e., embryos consistent with their expected stage of development, with blastomeres uniform in size, color and density, few irregularities or excluded cells and an intact and smooth zona pellucida were used. All embryos (with no sex determination) were thawed on ice and pools of 4 embryos at the same stage were prepared under a stereomicrosope (Table 1). Tubes containing embryos were manipulated for no more than 10 min at ambient temperature. After brief pelleting, the Tris-sucrose in excess was eliminated and tubes were stored at—80°C until proteomic analysis.

### 5.2 Nanoliquid Chromatography Coupled With Tandem Mass Spectrometry (nanoLC-MS/MS) Analysis

Proteins from pools of embryos were extracted and digested using the PreOmics iST-BCT kit following the manufacturer’s instructions. Briefly, samples were thawed and lysed (denatured, reduced and alkylated) for 10 min at 95°C then Trypsin/LysC digested for 60 min at 37°C. Purification of peptides was then carried out at room temperature on spin cartridge and peptides were finally eluted in 10 µL of LC-load buffer. The volume corresponding to one equivalent-embryo (corresponding to approximately 300 ng proteins) was then loaded on a 75 μm × 250 mm IonOpticks Aurora 2 C18 column (Ion Opticks Pty Ltd., Bundoora, Australia). Peptide analysis was performed as previously described (Banliat et al., 2020). A gradient of basic reversed-phase buffers (Buffer A: 0.1% formic acid, 98% H_2_O MilliQ, 2% acetonitrile; Buffer B: 0.1% formic acid, 100% acetonitrile) was run on a NanoElute HPLC System (Bruker Daltonik GmbH, Bremen, Germany) at a flow rate of 400 nL/min at 50°C. The liquid chromatography (LC) run lasted for 120 min (2–15% of buffer B during 60 min; up to 25% at 90 min; up to 37% at 100 min; up to 95% at 110 min and finally 95% for 10 min to wash the column). The column was coupled online to a TIMS TOF Pro (Bruker Daltonik GmbH, Bremen, Germany) with a CaptiveSpray ion source (Bruker Daltonik). The temperature of the ion transfer capillary was set at 180°C. Ions were accumulated for 114 ms, and mobility separation was achieved by ramping the entrance potential from −160 V to −20 V within 114 ms. The acquisition of the MS and MS/MS mass spectra was done with average resolutions of 60,000 and 50,000 full width at half maximum (mass range 100–1700 m/z), respectively. To enable the PASEF method, precursor m/z and mobility information was first derived from full scan TIMS-MS experiments (with a mass range of m/z 100–1700). The quadrupole isolation width was set to 2 and 3 Th and, for fragmentation, the collision energies varied between 31 and 52 eV depending on the precursor mass and charge. TIMS, MS operation and PASEF were controlled and synchronized using the control instrument software OtofControl 5.1 (Bruker Daltonik). LC-MS/MS data were acquired using the PASEF method with a total cycle time of 1.31 s, including 1 TIMS MS scan and 10 PASEF MS/MA scans. The 10 PASEF scans (100 ms each) containing, on average, 12 MS/MS scans per PASEF scan. Ion mobility-resolved mass spectra, nested ion mobility vs. m/z distributions, as well as summed fragment ion intensities were extracted from the raw data file with DataAnalysis 5.1 (Bruker Daltonik GmbH, Bremen, Germany).

### 5.3 Protein Identification and Data Validation

Peptides were identified using the MASCOT software (version 2.5.1; Matrix Science, 454 London, United Kingdom) against the UniProt *Bos taurus* database (May 2019; 23523 sequences) using its automatic decoy database search to calculate a false discovery rate (FDR). The parameters used for database searches included trypsin as enzyme (one missed cleavage allowed), carbamidomethylcysteine as fixed modification, oxidation of methionine and N-terminal protein acetylation as variable modifications. Monoisotopic mass was considered and mass tolerance was set at 15 ppm for MS ions and 0,05 Da for MS/MS ions. Mascot results from the target and decoy databases were incorporated to Scaffold Q+ software (version 5.0.1, Proteome Software, Portland, United States, www.proteomesoftware.com) for data validation. Threshold for peptide and protein identification were set to 95.0% as specified by the Peptide Prophet algorithm (Keller et al., 2002) and the Protein Prophet algorithm (Nesvizhskii et al., 2003). To exclude potential human contaminants from the pool of keratin proteins (KRT 1, 2, 5, 6A, 7, 8, 10, 14, 18, 19, 24, 28, and 75), the peptides identified in the *Bos taurus* database were blasted against the *Homo sapiens* homologous sequences. For all keratins except KRT14, the peptide sequences were specific to *Bos taurus*, meaning that except for KRT14, we were able to exclude human contaminants.

### 5.3 Label-Free Protein Quantification and Statistical Analysis

All proteins containing at least two unique peptides (FDR <0.01%) were considered for protein quantification. Protein quantification was based on a label-free approach using the spectral counting method, as previously described (Liu et al., 2004; Old et al., 2005). Scaffold Q+ software (Proteome Software, version 5.0.1) was used using the Spectral Count quantitative module. The normalization of spectra among samples was realized in Scaffold by adjusting the sum of the selected quantitative values for all proteins within each MS sample to a common value, which was the average of the sums of all MS samples present in the experiment. This was achieved by applying a scaling factor for each sample to each protein or protein group. Thus, numbers of normalized weighted spectra (NWS) were tabulated using experiment-wide protein clusters.

Statistical analysis was performed on proteins quantified with minimum 2 NWS on average in at least one stage. To obtain an overview of proteomic data, principal component analysis (PCA) of all samples was carried out using RStudio software (version 1.4.1106) and the FactoMineR and ggplot2 packages. Analysis of variance (ANOVA) on biological replicates was done using RStudio. The hierarchical clustering of differentially abundant proteins (DAPs; ANOVA’s p-value ≤ 0.05) were done using Spearman correlations and the gplots package of RStudio. Finally, pairwise comparisons in protein abundance were evaluated by Student’s t-tests using R. Proteins were considered as differentially abundant with a t-test p-value ≤ 0.05. A fold-change ratio ≥2 was retained for the functional analysis.

### 5.4 Functional Analysis of Differentially Abundant Proteins

The overlap of identified proteins between stages was visualized using the jvenn online tool (http://jvenn.toulouse.inra.fr) (Bardou et al., 2014). The functional analysis of DAPs in the two main clusters (ANOVA p-value ≤ 0.05) was performed using the Metascape online tool (metascape.org) (Zhou et al., 2019). The *Bos taurus* taxonomy is not available in Metascape therefore orthologs between *Bos taurus* and *Homo sapiens* were first searched using the HCOP orthology prediction tool (www.genenames.org/tools/hcop/). Human orthologs were found for all *Bos taurus* genes. The resulting gene lists without redundancy were used in the Metascape enrichment analysis using the *Homo sapiens* genome as background and the following ontology sources: KEGG (Kyoto Encyclopedia of Genes and Genomes) Functional Sets, KEGG Pathway, GO Biological Processes and Reactome Gene Sets. The cellular component enrichment analysis was carried out with the GO Cellular components source. Terms with a p-value < 0.01, a minimum count of 3, and an enrichment factor >1.5 (the enrichment factor is the ratio between the observed counts and the counts expected by chance) were collected and grouped into clusters based on their membership similarities. In histograms presenting the enriched terms, the most statistically significant term within a cluster is chosen by Metascape to represent the cluster.

The functions of DAPs in pairwise comparisons between stages (t-test p-value ≤ 0.05) considering a fold-change ratio ≥2 were further analyzed using the Proteomaps (proteomaps.net) and STRING (string-db.org, version 11.5) online tools (Liebermeister et al., 2014; Szklarczyk et al., 2015). Proteomaps graphics were generated from the gene lists of DAPs and related NWS values and using the *Homo sapiens* database as background, which is better annotated that those of *Bos Taurus* in Proteomaps. Proteomaps are built automatically from proteome quantitative data and based on the KEGG pathways gene classification. To create a Proteomap, the total area is first divided into polygons representing the top-level functional categories. These polygons are constructed from a Voronoi diagram, where the polygons’ areas were chosen to represent copy numbers weighted by protein chain lengths (the investment in terms of amino acids, also termed the mass fraction). The top-level areas are then subdivided into subcategories and the procedure is repeated down to the level of individual proteins. In the Proteomaps, functionally related proteins are arranged in common regions with similar colors. In addition, an enrichment analysis and protein-protein interaction (PPI) network were generated from each gene list of DAPs using STRING and the *Bos taurus* as organism background (which is well annotated in STRING). The settings for PPI networks were the full STRING network (in which the edges indicate both functional and physical protein interaction) as network type; a minimum required interaction score of 0.4; and the experiments, databases, co-expression, neighborhood, gene fusion and co-occurrence as interaction sources. The disconnected nodes in the network were discarded and only PPI networks with an enrichment p-value < 0.01, indicating that the proteins were at least partially biologically connected as a group, were retained. Finally, the STRING networks of DAPs together with their regulation pattern (up or downregulated) were imported into Cytoscape (version 3.9.0) in order to build interactions networks integrating those data (Shannon et al., 2003). The three most enriched KEGG pathways (with lowest FDR) identified by STRING were finally added in the networks.

## Data Availability

The datasets presented in this study can be found in online repositories. The names of the repository/repositories and accession number(s) can be found below: The datasets PXD030994 and 10.6019/PXD030994 for this study can be found in the ProteomeXchange Consortium *via* the PRIDE (Perez-Riverol et al., 2022) partner repository (www.ebi.ac.uk/pride/archive/login).

## Supplementary Material

Supplementary Figure S1 Representative pictures of the bovine embryos collected at the 4–6 cell, 8–12 cell, morula, compact morula and blastcyst stages.

Supplementary Table S1 Lists of proteins identified and quantified with at least two normalized weighed spectra (NWS) in pools of cattle embryos developed *in vivo*.

Supplementary Table S2 Classification of differentially abundant proteins (DAPs) according to the hierarchical clustering (ANOVA p-value ≤ 0.05) and Metascape enrichment analysis of upregulated (cluster 1) and downregulated (cluster 2) proteins during development.

Supplementary Table S3 Lists of differentially abundant proteins (DAPs) in each pairwise comparisons between stages (t-test p-value ≤ 0.05). The DAPs with fold-change ratios ≤0.5 or ≥2 are highlighted.

Supplementary Table S4 Functional enrichment analysis of DAPs between the 4–6 cell and 8–12 cell stages. The analysis was performed using STRING.

Supplementary Table S5 Functional enrichment analysis of DAPs between the 8–12 cell and morula stages. The analysis was performed using STRING.

Supplementary Table S6 Functional enrichment analysis of DAPs between morula and compact morula stages. The analysis was performed using STRING.

Supplementary Table S7 Functional enrichment analysis of DAPs between compact morula and blastocyst stages. The analysis was performed using STRING.
