# Supplementary figures and images for: Dynamic Changes in the Proteome of Early Bovine Embryos Developed In Vivo
Source: Front Cell Dev Biol. 2022 Mar 21;10:863700. doi: 10.3389/fcell.2022.863700 (PMC8979002; doi:10.3389/fcell.2022.863700)

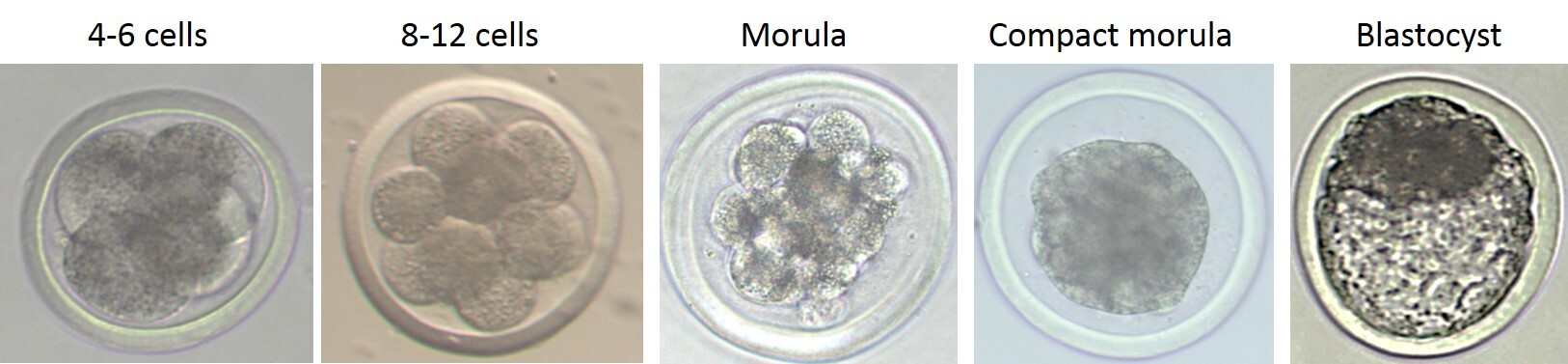

Supplement: Supplementary file 3 [file Image1.JPEG]
